# Mind Your Grip: Even Usual Dexterous Manipulation Requires High Level Cognition

**DOI:** 10.3389/fnbeh.2017.00220

**Published:** 2017-11-06

**Authors:** Erwan Guillery, André Mouraux, Jean-Louis Thonnard, Valéry Legrain

**Affiliations:** ^1^Institute of Neuroscience, Université catholique de Louvain, Brussels, Belgium; ^2^Department of Physical and Rehabilitation-Medicine, Saint-Luc University Hospital, Université catholique de Louvain, Brussels, Belgium; ^3^Psychological Sciences Research Institute, Université catholique de Louvain, Louvain-la-Neuve, Belgium

**Keywords:** dual-task, motor-cognitive interference, precision grip, grip-lift

## Abstract

Simultaneous execution of cognitive and sensorimotor tasks is critical in daily life. Here, we examined whether dexterous manipulation, a highly habitual and seemingly automatic behavior, involves high order cognitive functions. Specifically, we explored the impact of reducing available cognitive resources on the performance of a precision grip-lift task in healthy participants of three age groups (18–30, 30–60 and 60–75 years). Participants performed a motor task in isolation (M), in combination with a low-load cognitive task (M + L), and in combination with a high-load cognitive task (M + H). The motor task consisted in grasping, lifting and holding an apparatus instrumented with force sensors to monitor motor task performance. In the cognitive task, a list of letters was shown briefly before the motor task. After completing the motor task, one letter of the list was shown, and participants reported the following letter of the list. In M + L, letters in the list followed the alphabetical order. In M + H, letters were presented in random order. Performing the high-load task thus required maintaining information in working memory. Temporal and dynamic parameters of grip and lift forces were compared across conditions. During the cognitive tasks, there was a significant alteration of movement initiation and a significant increase of grip force (GF) throughout the grip-lift task. There was no interaction with “age”. Our results demonstrate that planning and the on-line control of dexterous manipulation is not an automatic behavior and, instead, that it interacts with high-level cognitive processes such as those involved in working memory.

## Introduction

Dexterous manipulation is probably the most habitual way we interact with objects of the environment. Precision grip movements, besides their apparent simplicity, rely on complex anticipatory and online controls on the movement (Westling and Johansson, 1984; Nowak and Hermsdörfer, 2004; Witney et al., 2004). The great majority of studies aiming at understanding the neural mechanisms underlying dexterous manipulation have focused on motor tasks performed in isolation. Similarly, assessment of motor dysfunction in patients, or assessment of changes in motor function induced, for example, by post-stroke rehabilitation, always rely on the performance of isolated motor tasks. However, in our daily life, object manipulation is most often performed concomitantly to other tasks, such as manipulating a glass while having a conversation, or interacting with a computer or smartphone. Goal-directed motor actions then require focusing or sharing attention on relevant stimuli in order to minimize detrimental effects from irrelevant distracters. High-level cognitive control is therefore needed to allow efficient achievement of relevant actions. Such questions were investigated, among other procedures, by the dual-task paradigm, an experimental procedure that requires performing two tasks simultaneously in order to compare performance between dual-task and single-task conditions (Kahneman, 1973; Abernethy, 1988; Pashler, 1994). If the two tasks interfere with each other when performed simultaneously, it is assumed that both tasks share similar resources and processing abilities. Accordingly, recent studies have shown that performing a high-order cognitive task may interfere with the realization of upper limb movements such as reaching and grasping (Li et al., 2009; Spiegel et al., 2013, 2014; Gunduz Can et al., 2017), precision grip-lift (Guillery et al., 2013; Bumsuk et al., 2014), precision and power grip squeezing (van Dijck et al., 2015), force tracking (Au and Keir, 2007; Voelcker-Rehage et al., 2006; Mehta and Agnew, 2011; Temprado et al., 2015), and tapping (Serrien, 2009; Fraser et al., 2010; Korotkevich et al., 2015). These studies suggest that habitual dexterous manipulation may not only rely entirely on automatic processes and, instead, may involve or interact with higher-order cognitive functions to be planned and executed.

In a recent study, we aimed to test this hypothesis by means of a dual-task paradigm combining a simple object manipulation with a high-level cognitive task (Guillery et al., 2013). The experimental procedure consisted in instructing participants to perform two tasks simultaneously or in isolation in order to assess how the two tasks impact each other when performed in combination. The motor task consisted in manipulating an apparatus equipped with force sensors to measure the forces applied while gripping, lifting and maintaining the object for a short duration. The cognitive task consisted in performing a complex visual search and counting task. As compared to when the motor task was performed in isolation, when participants performed the dual task, they took more time to assess the physical properties of the manipulated object during the preload phase, and applied more grip force (GF) when holding the object. This observation supports the view that simple object manipulation is not a strictly automatic behavior and, instead, that at least some aspects of its achievement involves higher-order cognitive functions. However, the cognitive task did not allow determining which high-order processes were involved in the observed interference. Most importantly, the visual search and counting task involved motor activities such as head and eye movements. Therefore, the differences observed in the dual-task condition could have resulted from a competition of resources specifically involved in motor control.

In the present study, we explore further the involvement of high-level cognitive processes on dexterous manipulation by combining a motor grip-lift task with a working memory task minimizing the involvement of motor control and motor imagery. Besides its role in the short-term storage of sensory inputs (Collette et al., 2007), working memory is also regarded as an executive function involved in monitoring control over information processing (Hegarty et al., 2000) such as prioritizing the processing of relevant target stimuli from interfering distracters (Lavie and De Fockert, 2005). Working memory contributes to optimizing attention by keeping a memory trace of the features of task-relevant target stimuli during the achievement of target detection tasks (Desimone and Duncan, 1995) and by protecting task execution from the intrusion of distracter stimuli (Lavie, 2010). Studies have shown indeed that, during dual task, participants’ performance in the primary task are more sensitive to distraction when high working memory load is used by a second unrelated task (de Fockert et al., 2001; Lavie et al., 2004; Lavie and De Fockert, 2005). On the contrary, the ability to control detrimental effects from distracters on task achievement is increased when participants are encouraged to used working memory abilities to the perform the primary task (Berti and Schröger, 2003; SanMiguel et al., 2008; Legrain et al., 2011a,b, 2013).

Here, participants were requested to perform the grip-lift motor task either in isolation or in combination with one of two cognitive tasks contrasting only by how they recruited working memory. In both dual task conditions, the execution of the motor task was preceded by the presentation of a string of letters. After the execution of the grip-lift movement, a single letter was presented and participants were asked to report which letter of the initial string was following the target letter. In a low-load condition, the letters of the initial string were presented in alphabetic order. Hence, participants could rely simply on their semantic knowledge about the Latin alphabet to perform the cognitive task. In a high-load condition, the letters were presented in a pseudo-random non-alphabetic order. Hence, performing the cognitive task required participants to store and rehearse in working memory the complete list of letters in their correct order while they executed the grip-lift movement. The two cognitive conditions were therefore matched according to sensory inputs and response requirement but contrasted according to working memory load. Our hypothesis was that if some aspects of the grip-lift movement are under the control of high-level cognitive process, they should be impacted when these cognitive processes are captured by the second task. In other words, we postulated that, as compared to a condition during which the motor task is executed alone, some dynamic and temporal parameters of the grip-lift movement would be changed when the motor task is achieved concomitantly to a cognitive task, especially in the high load working memory condition. To minimize the contribution of inter-individual differences in working memory abilities, the length of the strings of items to memorize during working memory tasks was adapted to each participant. As it is known that age can impact both motor and cognitive functions (Maylor, 1998; Cole et al., 1999), the potential effect of this variable was tracked by splitting the participant sample in three separate age groups.

## Materials and Methods

### Participants

Thirty-seven healthy volunteers took part in the experiment (19 men and 18 women, mean age: 45 ± 19, range: 21–78). Participants were assigned to one out of three groups according to their age: *Young* (from 20 years to 30 years old: *n* = 13, mean age = 26 ± 2), *Middle Age* (from 30 to 60: *n* = 12, mean age = 43 ± 9) and *Older* (from 60 and more: *n* = 12, 68 ± 6). All participants had normal or corrected to normal vision, and no history of a neurological disease. The experimental procedure was approved by the local ethic committee (Comité d’Ethique hospitalo-facultaire, Cliniques Universitaires Saint-Luc & Université catholique de Louvain) in agreement with the latest version of the Declaration of Helsinki, and all participants signed a consent form prior to the experimental session. Participants received financial compensation.

### Pre-Experimental Assessment

Before the actual experiment, the cognitive abilities of the participants were tested with the Mini-Mental State Examination (MMSE; Folstein et al., 1975). Handedness was tested with the Edinburgh Oldfield Handedness Inventory (Oldfield, 1971). Most importantly, the pre-experimental evaluation included an assessment of memory span for letters, i.e., the capacity of working memory to rehearse series of letters for their immediate use (Conway et al., 2005). This was achieved in order to individually adjust the working memory task of the experimental session to the abilities of the participant and, thereby, avoid using series of items beyond working memory capacity. The assessment of working memory span was similar to the standard Digit Span subtest of the Wechsler Memory Scale (Wechsler, 1981). Participants were asked to memorize a series of consonant letters presented on a screen during 2.25 s and to repeat the entire series in the correct order immediately after they disappeared from the screen. Vowels were not used in order to prevent participants from chunking the letters into pronounceable words or pseudo-words. Meaningful sequences such as acronyms were also avoided. Trials started with a series of three letters. After each repetition of two successful trials, one item was added to the series. Otherwise, the procedure was stopped. The length of the longest series a participant could repeat successfully was considered as his memory span of letters.

### Stimuli and Apparatus

The apparatus used to perform the grip-lift task was a 275 g, 108 × 56 × 38 mm (height, width and depth) mechanical assembly (fMRI-GLM, Arsalis, Belgium). The device was instrumented with full Wheatstone Bridges incorporating three strain gauges load sensors allowing to measure the force perpendicular to each contact surface (GF left and GF right) as well as the tangential force applied on the object (load force, LF; Guillery et al., 2013). It was calibrated up to a full scale of 30 N in each direction and demonstrated a maximum nonlinearity of 0.70% for LF and 0.35% for GF. The analog signals were amplified, filtered with a Bessel 4-pole 150 Hz low-pass filter and sampled at 2000 Hz with a resolution of 16 bits. The resolution of the force measurements was 0.002 N for GF and 0.001 N for LF. The data was stored on a personal computer for offline analysis.

Visual and auditory stimuli were generated using Matlab 7 (The MathWorks Inc., MA, USA) and the Cogent 2000 graphics toolbox^1^. Visual material and instructions were displayed on a 17 inch LCD monitor (AL1703sm, Acer, Taiwan) positioned approximately 1 m in front of the participant, using a 800 × 600 resolution and a 60 Hz refresh rate. The visual stimuli consisted in a list of consonants, a consonant or a cross displayed at the center of the screen in capital letters (font: Tahoma, size: 80). They encompassed approximately 3° of visual angle. The auditory stimulus was a 725 Hz tone lasting 0.19 s.

### Procedure

Participants sat comfortably in a chair in front of a desk supporting the apparatus and LCD monitor. During the experiment, they were instructed to keep their non-dominant hand at rest, and their dominant hand around the apparatus. Instructions about the task were given verbally. They were asked to perform the different tasks under three conditions. In the first condition (M), participants performed a motor task without any concomitant cognitive task. In the second and third conditions, participants concomitantly performed the same motor task with either a low-load cognitive task (M + L) or a high-load cognitive task (M + H). They were asked to perform the cognitive tasks as fast and as accurately as possible. In the dual-task conditions they were asked not to give priority to the motor or to the cognitive tasks. Participants were given the opportunity to practice the tasks before the experiment in five consecutive trials for each condition. Next, participants performed six experimental blocks of 8 trials each, two blocks per condition. The order of the conditions was counterbalanced across participants.

**Figure 1:**
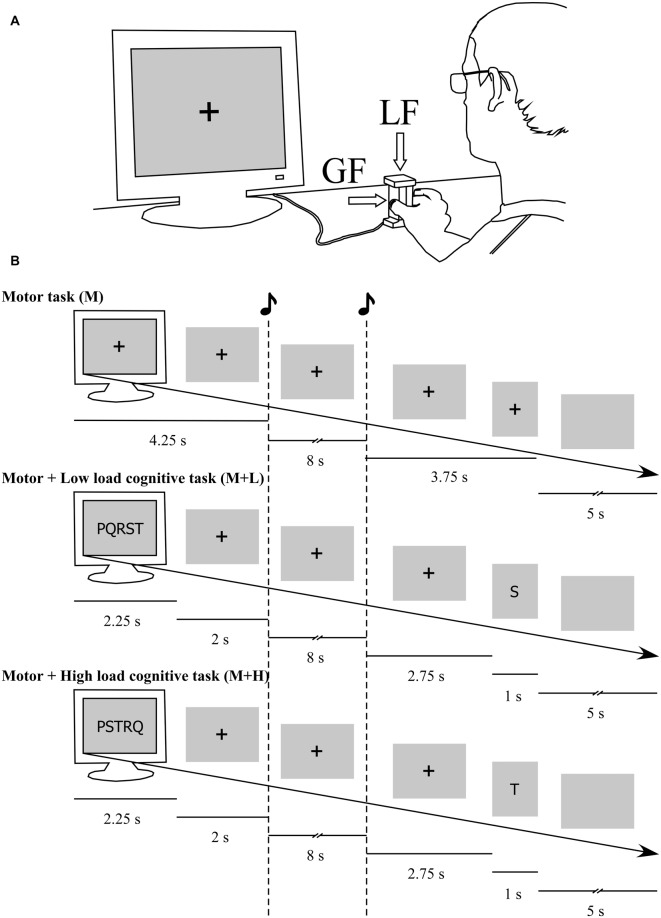
Experimental procedures and time course of trials in the different conditions. **(A)** Participants were seated in front of a computer display and grasped the apparatus between the thumb and the index. The device was equipped with strain gauges which allowed measuring the grip force (GF) and load force (LF) developed during the experiment. They had to keep their eyes on the fixation cross when no other stimulus was displayed. **(B)** In the Motor condition (M), an auditory tone prompted the participant to grip, lift and hold the object approximately 5 cm above the table. After 8 s, a second auditory tone prompted the participant to put down the object on the table and to reposition their hand at rest next to the object. In the Motor + Cognitive conditions, a list of letters appeared at trial onset. Participants were requested to remember this list of letters, whose length was adapted to their individual memory span. The list was in alphabetical order in the Motor + Low-load cognitive condition (M + L) and in randomized order in the Motor + High-load cognitive condition (M + H). Participants performed the grip-lift-hold task while maintaining the list of letters in working memory. After the motor task, a random letter from the initial memory set reappeared, and participants were instructed to report verbally which letter of the memory set was presented after that letter.

The motor task (M) consisted in a grip-lift movement (Figure 1A; Westling and Johansson, 1984; Guillery et al., 2013). The trial started with the participants fixating a black cross positioned at the center of the screen. After 4.25 s an auditory tone prompted the participants to grip, lift and maintain the apparatus approximately 10 cm above the table. A second auditory tone, occurring 8 s after the onset of the first, prompted the participants to put down the apparatus on the table and to reposition their hand at rest around the apparatus. Five seconds separated the end of each trial from the beginning of the following trial. This ensured that participants had enough time to reposition their hand next to the apparatus (Figure 1B).

In the M + L and M + H conditions, each trial started with the presentation of a memory set consisting of a list of letters on the screen. The letters were chosen among the following list of letters: J, K, L, M, N, P, Q, R, S, T, V. The length of the set was adapted individually to match the length of the memory span for letters assessed before the beginning of the experiment. The length of the memory set was then fixed for the whole experimental session. The letters were shown for 2.25 s, and then replaced by a fixation cross (Figure 1B). Such as in the M condition, an auditory tone was presented 4.25 s after trial onset, prompting the participant to perform the motor task. Then, after 8 s, the second auditory tone occurred, prompting the participant to put down the apparatus on the table. Finally, 2.75 s after the second auditory tone, one single letter belonging to the list was presented for 1 s, and participants were instructed to report verbally which letter followed that letter in the memory set presented at the beginning of the trial.

In the M + L condition, the letters of the memory set were presented in alphabetic order (e.g., JKLMN). Hence, participants could rely simply on their semantic knowledge about the Latin alphabet. In the M + H condition, the letters were presented in a pseudo-random non-alphabetic order. Hence, performing the task required participants to store and rehearse in working memory the complete list of letters in their correct order while they performed the motor task. String of the same order was never repeated.

### Coefficient of Friction

The amount of GF required to lift the object without letting it slip through the fingers is dependent on the coefficient of friction between the fingers and the object surface. Therefore, before and after the experiment, we estimated the skin-apparatus coefficient of static friction (CF) by asking participants to perform a series of eight lift-and-drop maneuvers with the apparatus. The participants lifted and held the instrument stationary, then gradually released the grip until the object slipped due to gravity (Johansson and Westling, 1984). For each lift-and-drop maneuvers, the static CF was estimated as half the LF/GF ratio at slip onset (Johansson and Westling, 1984).

### Measures

The performance of the cognitive tasks was assessed by computing the percentage of correct answers, i.e., the percentage of trials for which the correct letter of the memory set was reported.

**Figure 2:**
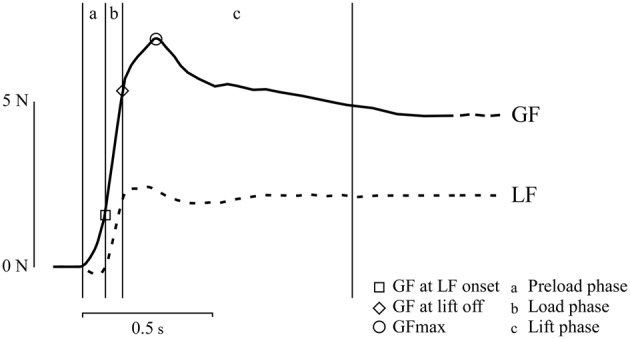
Time course of GF (continuous trace) and LF (dotted trace) during a typical trial of the grip-lift-hold task. The Preload phase (a) corresponds to the time separating the first contact of one finger on the apparatus and the onset of a positive LF. The Load phase (b) corresponds to the time during which a parallel increase of GF and LF is observed. The Lift phase (c) corresponds to the time during which the apparatus is raised and stabilized over the table. Several force measures were extracted from the waveforms. The GF at LF onset corresponds to the value of GF at the beginning of the Load phase. The GF at lift-off corresponds to the value of GF when LF equals the weight of the apparatus, i.e., when the apparatus begin its lift-off. The GF max corresponds to the maximum value of GF. Values are in Newton (N) and second (s).

Forces (in N) were analyzed using Matlab 7.5 (The MathWorks, Inc., MA, USA). Signals were low-pass filtered using a Butterworth filter (cut-off: 15 Hz; slope: 2 dB). Typical GF and LF traces are shown in Figure 2 (Guillery et al., 2013). In the M + L and M + H conditions, trials for which the participants did not provide the correct answer in the cognitive task were rejected from further analyses.

The time course of the grip-lift movement can be decomposed into series of distinct phases (in ms): the Preload phase, the Load phase, the Lift phase and the Hold phase (see, Guillery et al., 2013) for more details about these measures). The *Preload phase* is defined as the time between initial contact of the fingers with the apparatus and the onset of the (LF > 0.1 N), i.e., when participants start to apply an upward vertical force to load the object. The *Load phase* is defined as the time during which both GF and LF increase up to the instant when LF equals the weight of the apparatus (2.75 N), i.e., the instant when the apparatus is actually lifted from the table. The *Lift phase* is defined as the time interval during which the apparatus is lifted upwards until stabilization above the table. Finally, the *Hold phase* is defined as the time interval during which the apparatus remains stable above the table.

In addition to assessing the duration of these phases, the GF and LF forces measured at different time points were used to summarize the performance of the motor task: GF at LF onset, GF at lift off, GF max and GF hold. The *GF at LF onset* and the *GF at lift off* provide information about the GF applied at the very early stage of the movement, i.e., at the beginning and the end of the Load phase, respectively. The *GF max* corresponds to the maximum GF applied during the trial, and usually occurs at the end of the Lift phase. The *GF hold* is computed by averaging the GF during the Hold phase, and provides information about how the GF is maintained while the apparatus remains stable above the table. The GF hold was also used to compute the Safety Margin (SM). The SM provides information on the excess of GF applied onto the object to prevent it from slipping through the fingers. It is expressed as the ratio between the excess of GF while holding the object (GF hold − GF at slip), divided by the GF hold.

Finally, to assess the effect of the cognitive task on the temporal coupling of load and GFs, a cross-correlation analysis was performed using the first derivative of LF (dLF/dt) and the first derivative of GF (dGF/dt), in the time-interval separating the first contact with the apparatus and the GF max (Duque et al., 2003). The time lag at maximum correlation provides an estimate of the time lag between the two signals and the maximum coefficient of correlation provides an estimate of the similarity between the two time courses. We computed the absolute value of time lags.

### Statistical Analyses

Statistical analyses were performed using SPSS 21 (IBM, NY, USA). For all analyses, the significance level was set a *p* ≤ 0.05.

The memory span of letters was compared between the different groups using an analysis of variance (ANOVA) with “age” (*young* vs. *middle aged* vs. *older*) as a group factor.

The impact of the cognitive tasks on the performance of the motor task (M vs. M + L vs. M + H) was assessed by comparing the different temporal and force measures obtained from the grip-lift task using an ANOVA with “condition” as within-subject factor and “age” as between-subject factor. Values of CF obtained before and after the experiment were compared using the same ANOVA. Greenhouse-Geisser corrections for degrees of freedom and contrasts analyses with Student *t*-tests were used when necessary (non-corrected for multiple comparisons). Effect sizes were estimated by means of partial eta squared and sensitivities were estimated by observed power.

Finally, to compare the time courses of GF during the grip-lift task performed in the different conditions, single-subject average waveforms of GF obtained for each of the three conditions, aligned relative to lift off, were compared using a point-by-point ANOVA with “condition” as within-subject factor and “age” as between-subject factor. This method allowed identifying the time intervals where the GF traces showed a significant main effect of “condition” (M vs. M + L vs. M + H) and “age”, or a significant interaction between the two factors. When significant, the same method was applied to compare the different average waveforms of GF of each condition pairwise, using point-by-point *t*-tests.

## Results

### Participants

According to the Edinburgh Oldfield Handedness Inventory (Oldfield, 1971), 34 participants were right-handed and three participants were left-handed. Their score at the MMSE was 29.97 ± 0.16 (mean ± standard deviation (SD)). This indicates the absence of any blatant cognitive deficit. Statistical analyses of the memory span of letters did not reveal any significant difference between age groups (*p* = 0.7). The mean span of letters was 7 ± 1 in each group. Consequently, the mean length of letter strings used in the M + L and M + H tasks was identical in all groups.

### Performance of the Cognitive Task

The participants provided the correct answer to the cognitive task in 98 ± 5% of trials in the M + L condition and 77 ± 13% of trials in the M + H condition. The difference in the percentage of errors between the M + L and M + H conditions was significant (*F*_(1,6)_ = 47.7; *p* < 0.001). The performances were not affected by “age” (*p* = 0.6).

### Performance of the Motor Task

**Figure 3:**
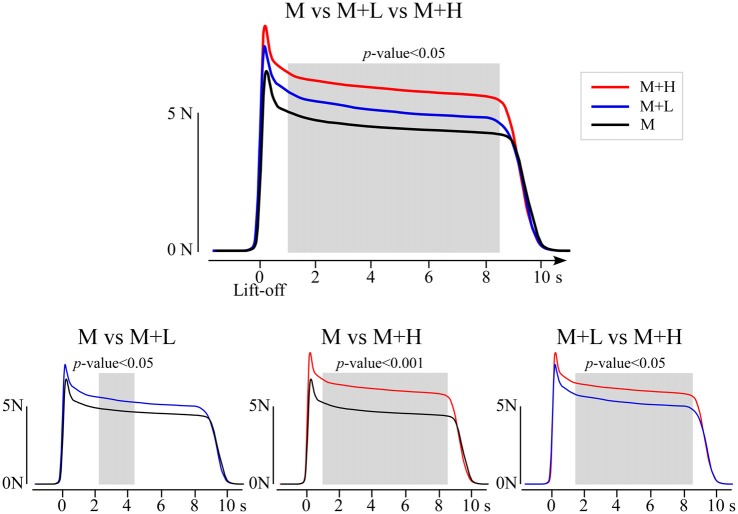
Group-level average waveforms of GF obtained in each of the three conditions, after realigning trials to the lift-off of the object. The waveforms were compared using a point-by-point repeated-measures analysis of variance according to the conditions. This analysis revealed a significant difference across the three conditions extending between 0.98 s and 8.66 s after lift-off. *Post hoc* point-by-point t-tests showed that GF was greater in the M + L than in the M conditions from 2.25 s to 4.45 s, greater in the M + H than in the M conditions from 0.98 s to 8.64 s, and greater in the M + H than in the M + L conditions from 1.52 s to 8.68 s.

**Figure 4:**
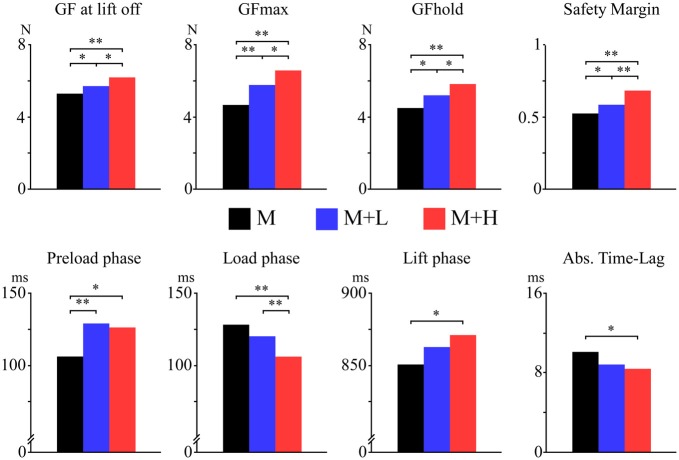
Pairwise comparisons of the different measures of motor performance in the M (black bars), M + L (blue bars) and M + H (red bars) conditions. A single asterisk indicates a *p*-value < 0.05, double asterisks indicate a *p*-value < 0.001.

Table 1 presents the group-level mean and SD of each measured parameter of the grip-lift task performed in the M, M + L and M + H conditions, and the main effects of the factor “condition”. The time courses of GF across the three conditions is illustrated at Figure 3. Figure 4 illustrates the pairwise comparisons between the three conditions.

**Table 1:** Mean and standard deviation (SD) of the parameters of the grip-lift task performed in the M, M + L and M + H conditions; and main effect of condition on these parameters.

Temporal parameters	M	M + L	M + H	Condition_Main effect of repeated measure ANOVA_				
	Mean (SD)	Mean (SD)	Mean (SD)	*f*	*df*	*p*-value	*η*^2^	Observed power
Preload phase (ms)	106 (46)	129 (60)	126 (56)	7.95	(2, 62)	<0.001	0.20	0.95
Load phase (ms)	128 (35)	120 (27)	106 (22)	13.68	(1.7, 44.8)	<0.001	0.31	0.99
Lift phase (ms)	851 (44)	863 (37)	871 (39)	4.73	(1.6, 50.7)	0.02	0.13	0.71
Cross-correlation coefficient	0.81 (0.07)	0.82 (0.06)	0.82 (0.06)	1.97	(2, 62)	0.15	0.06	0.39
Absolute time-lag (ms)	10.63 (8.09)	9.30 (8.61)	8.78 (7.13)	3.87	(2, 62)	0.03	0.11	0.68
Force parameters								
GF at LF onset (N)	1.60 (1.52)	1.60 (1.59)	1.79 (1.78)	1.90	(1.8, 56.2)	0.16	0.06	0.36
GF at lift-off (N)	5.27 (2.49)	5.68 (2.77)	6.17 (2.93)	8.94	(2, 62)	<0.001	0.22	0.97
GF max (N)	6.67 (2.7)	7.78 (3.68)	8.59 (4.0)	13.99	(2, 62)	<0.001	0.31	1.00
GF hold (N)	4.48 (1.99)	5.17 (2.96)	5.80 (2.95)	11.44	(15.4, 59.2)	<0.001	0.27	0.99
Safety margin	0.52 (0.21)	0.58 (0.17)	0.63 (0.14)	21.42	(2, 62)	<0.001	0.41	1.00

*Effect sizes were estimated by means of partial eta squared values and sensitivity were estimated by observed power. Values are in Newton (N) and second (ms)*.

#### Temporal Parameters

There was a significant main effect of “condition” on the duration of the Preload phase (*F*_(2,62)_ = 7.95; *p* < 0.001), the duration of the Load phase (*F*_(1.7,44.8)_ = 13.68; *p* < 0.001), and the duration of the Lift phase (*F*_(1.6,50.7)_ = 4.73; *p* < 0.02), indicating that the duration of the different phases of the grip-lift task were affected by the cognitive tasks. *Post hoc* comparisons showed that, as compared to the M condition, the Preload phase was significantly longer in the M + L condition (mean Δt = 23 ms; *t*_(36)_ = 3.73; *p* = 0.001) and the M + H condition (mean Δt = 20 ms; *t*_(36)_ = 3.25; *p* = 0.003). There was no difference in the duration of the Preload Phase between the M + L and M + H conditions (*t*_(36)_ = 0.48; *p* = 0.63). Conversely, the duration of the Load phase was significantly shorter in the M + H condition as compared to the M condition (mean Δt = 22 ms; *t*_(36)_ = 5.08; *p* < 0.001) and significantly shorter in the M + H condition as compared to the M + L condition (mean Δt = 14 ms; *t*_(36)_ = 5.95; *p* < 0.001). There was no significant difference between the M and M + L conditions (*t*_(36)_ = 1.75; *p* = 0.09). The duration of the Lift phase was significantly greater in the M + H condition as compared to the M condition (mean Δt = 20 ms; *t*_(36)_ = 2.21; *p* = 0.03), but there was no significant difference between the M and M + L conditions (*t*_(36)_ = 1.95; *p* = 0.06) and between the M + L and M + H conditions (*t*_(36)_ = 1.35; *p* = 0.19). Regarding the group factors, there was a significant main effect of “age” on the duration of the Preload phase (*F*_(2,31)_ = 3.78, *p* = 0.03), whose duration tended to be significantly shorter in the *middle age* group as compared to the *older* group (mean Δt = 52 ms; *t*_(16.8)_ = 2.07; *p* = 0.05). There was no interaction between the factors “condition” and “age” (*p* = 0.15).

#### Force Parameters

The cognitive tasks also exerted an effect on the GF applied while performing the motor task. There was a significant main effect of “condition” on the GF at lift-off (*F*_(2,62)_ = 8.94; *p* < 0.001), the GF max (*F*_(2,62)_ = 13.99; *p* < 0.001), and the GF hold (*F*_(15.4,59.2)_ = 11.44; *p* < 0.001; see Table 1 and Figure 4). *Post hoc* comparisons showed that the GF at lift-off was marginally greater in the M + L condition as compared to the M condition (mean Δ = 0.41 N; *t*_(36)_ = 2.17; *p* = 0.04), and significantly greater in the M + H as compared to the M condition (mean Δ = 0.92 N; *t*_(36)_ = 3.98; *p* < 0.001), and in the M + H as compared to the M + L condition (mean Δ = 0.49 N; *t*_(36)_ = 2.40; *p* = 0.02). This was also the case for the GF max and the GF hold, which were both significantly greater in the M + L than in the M conditions (GF max: mean Δ = 1.11 N; *t*_(36)_ = 3.55; *p* = 0.001; GF hold: mean Δ = 0.69 N; *t*_(36)_ = 2.45; *p* = 0.019), in the M + H than in the M conditions (GF max: mean Δ = 1.92 N; *t*_(36)_ = 5.20; *p* < 0.001; GF hold: mean Δ =1.32 N; *t*_(36)_ = 4.41; *p* < 0.001) and in the M + H than in the M + L conditions (GF max: mean Δ = 0.81 N; *t*_(36)_ = 2.59; *p* = 0.01; GF hold: mean Δ = 0.63 N; *t*_(36)_ = 2.4; *p* = 0.002). Other “condition” and “age” effects and their interactions were not significant (all *F* < 1.4; all *p* > 0.15).

There was no effect of “time” (before vs. after) or of “age” on the measures of the CF (all *F* < 0.45; *p* > 0.5). Therefore, for each participant, the SM was computed using the average of the CF measures performed before and after the experiment. The SM was significantly different between conditions (*F*_(2,62)_ = 21.42; *p* < 0.001). The SM was significantly increased in the M + L condition as compared to the M condition (mean Δ = 0.06; *t*_(36)_ = 3.39; *p* = 0.002), in the M + H as compared to the M condition (mean Δ = 0.11; *t*_(36)_ = 5.87; *p* < 0.001), and in the M + H as compared to the M + L condition (mean Δ = 0.05; *t*_(36)_ = 3.72; *p* = 0.001). Other “condition” and “age” effects and their interactions were not significant (all *F* < 2.6, all *p* > 0.08).

#### Temporal Coupling of Load and Grip Forces

The cross-correlation analysis between the time course of GF and LF showed a significant main effect of “condition” on the absolute time lag between GF and LF (*F*_(2,62)_ = 3.87; *p* < 0.03). The lag was significantly greater in the M condition as compared to the M + H condition (mean Δ = 1.85 ms; *t*_(36)_ = 2.67; *p* = 0.01), but there was no significant difference between M and M + L conditions (mean Δ = 1.33 ms; *t*_(36)_ = 1.79; *p* = 0.08) and between M + L and M + H conditions (mean Δ = 0.52 ms; *t*_(36)_ = 0.90; *p* = 0.37). There was also a main effect of “age” on the lag (*F*_(2,30)_ = 5.95; *p* < 0.01). The absolute time-lag was greater in the *older* group as compared to the *young* group (mean Δ = 8.35 ms; *t*_(14.5)_ = 2.67; *p* = 0.02) and the *middle aged* group (mean Δ = 8.15 ms;* t*_(13.7)_ = 2.65; *p* = 0.02). There was no significant effect of “condition” on the coefficient of cross-correlation. For all the parameters of the grip-lift task, the factor “age” never interacted significantly with the factor “condition” (all *F* < 1.4; all *p* > 0.2).

#### GF Time Courses

The point-by-point ANOVA showed a significant difference in the time courses of GF across the different conditions (Figure 3), extending between 0.98 s and 8.66 s after lift-off (*p* = 0.002). There was no effect of the factors “age”. Pairwise comparisons of GF time courses showed that the GF was significantly increased from 2.25 s to 4.45 s in the M + L as compared to the M condition, from 0.98 s to 8.64 s in the M + H as compared to M conditions, and from 1.52 s to 8.68 s in the M + H as compared to M + L conditions (Figure 3).

## Discussion

In the present study, we show that performing a simple motor task such as gripping, lifting and holding an object can be impacted by the concomitant performance of a cognitive task. Several aspects of the grip-lift motor performance were modified during the dual task conditions as compared to the condition during which the motor task was performed alone, especially during the high load working memory condition. This indicates that some aspects of basic motor control involve high-order cognitive processes. Importantly, as compared to previous studies (Glover et al., 2004; Voelcker-Rehage and Alberts, 2007; Voelcker-Rehage et al., 2006; Li et al., 2009; Hesse and Deubel, 2011; Guillery et al., 2013; Behmer and Fournier, 2014; Bumsuk et al., 2014; Kawagoe and Sekiyama, 2014; Quak et al., 2014; Spiegel et al., 2014; Korotkevich et al., 2015; Maes et al., 2015; van Dijck et al., 2015), the performance of the cognitive tasks used in the present study did not involve any explicit motor function. For this reason, the cognitive-motor interaction observed in the present study cannot be due to competition for resources specifically involved in motor control. In addition, we used two conditions for the cognitive task contrasting only by the involvement of working memory. Studies using similar working memory paradigms have shown that working memory acts on attentional control by shielding task-relevant information from distracters (de Fockert et al., 2001; Berti and Schröger, 2003; Lavie et al., 2004; Lavie and De Fockert, 2005; SanMiguel et al., 2008; Legrain et al., 2011a,b, 2013). Importantly the set of items to memorize during the high-load cognitive task was adapted to each participant. Additionally, the memory span, and, consequently, the size of the memory set were not different between the three groups of age. The participants performed therefore the cognitive tasks with the same materials.

The most striking effect of the cognitive task on motor performance was that participants increased the GF applied against the object as soon as they started to lift the object and during the whole holding phase (GF at lift-off, GF max and GF hold, see Figure 4). This increase in GF was more pronounced in the high working memory load condition as compared to the low-load condition. The results show that the force that should be applied to lift and hold the object are adapted according to availability of cognitive resources, suggesting that adjustment of GF during these steps is under the control of high order cognitive functions.

It is worth noting that performing a demanding cognitive task can induce autonomic changes leading to a modification of fingertip moistness. Because skin moistness is known to markedly influence the coefficient of friction (André et al., 2010), it was crucial to determine whether the increased GF in the dual-task conditions was not merely a compensation for a reduction of friction between the fingertip and the manipulated object. For this reason, we measured the coefficient of friction of each participant, and found that there was no difference in the coefficients friction measured across trials, conditions and groups of age. As the increase in GF while performing the cognitive tasks was not compensating a reduction of friction, this increase led to an increase in the SM, i.e., the amount of excess force applied against the object to prevent it from slipping through the fingers. Increasing the SM implies increasing the muscular effort required to perform the motor task, and increasing the risk of damaging the manipulated object if it is delicate. Conversely, increasing the SM reduces the risk of slippage due to variations in GF, or to the occurrence of an external perturbation. When participants performed the cognitive tasks, the fluctuations in GF during the holding phase were not greater than when they performed the motor task alone, indicating that the cognitive task did not affect the constancy of the GF. Hence, the risk of slippage due to variations in GF was not increased in the dual task conditions. A possible explanation for the increased SM in these conditions could thus be that it reduced the need to monitor the environment for possible perturbations that could interfere with the object manipulation and require to adjust the GF (Westling and Johansson, 1984), or to monitor somatic input from the fingertip that could signal slippage of the object (Johansson and Westling, 1987), such as the afferent input that might be generated during partial slip (Johansson and Westling, 1987).

The cognitive tasks also had an effect on several temporal parameters of the motor task. First, there was an increase in the duration of the Preload phase in both dual task conditions, and an increase in the duration of the Lift phase in the high-load condition. An increase of the Preload phase duration had already been observed in our previous study (Guillery et al., 2013). Both of these phases of the grip-lift task are thought to reflects periods during which motor control operates based on feedback mechanisms integrating mechanical and proprioceptive inputs in order to optimize the contact of the fingers with the object and its stabilization after lift off Johansson and Westling (1984). The finding that more time is required for these phases in the dual task conditions suggests that these feedback mechanisms involve high-order cognitive processes. Conversely, the duration of the Load phase was reduced during the high-load condition. A possible explanation for the increased speed of this movement phase could be that, during the high-load condition, it is performed in a less feedback-controlled fashion. Another explanation could be that participants reduce the duration of this phase of the movement to reduce its interference on the performance of the working memory task.

The present study has some limitations. Unlike the low-load condition, the high-load condition required to use working memory functions. Working memory is a complex concept encompassing different definitions (Engel et al., 1997; Miyake et al., 2000). Whereas some authors consider working memory just as the transient activation of information contained in long-term declarative memory systems (Cowan, 1995), others have defined working memory as a separate system made of several subcomponents (Baddeley, 2003). From the present data, it is not possible to characterize specifically which of these components of working memory contributed to impact motor task parameters. In addition, it could be argued that such impact was observed because performing the working memory task engaged covert articulatory processes involved in rehearsing verbal information. Indeed, it has been shown that verbal rehearsal is based on motor abilities (Hall et al., 1997; Saimpont et al., 2013) and generates activity in primary sensorimotor cortices (Lotze et al., 2000; Nota and Honda, 2004). Therefore, the results of our study cannot exclude that at least part of the observed effects were due to competition for motor processes engaged both by the motor task and by covert verbal rehearsal during the working memory task. Further studies will be needed to characterize more specifically which cognitive processes are involved in the regulation of motor behavior as that here manipulated.

To conclude, mimicking the everyday activity of fine-grip object manipulation, our experiment confirms that the performance of an apparently simple and automatic motor activity of grasping and lifting an object is under the influence of higher-order cognitive processes. Several previous studies have advocated the clinical usefulness of assessing the performance of a grip-lift task to evaluate motor function in patients (Berner et al., 2007; Johansson and Flanagan, 2009). However, these clinical assessments are always performed in isolation. Assessment of the dynamic and temporal parameters of the grip-lift task in a dual-task paradigm could provide a better-suited tool to assess motor performance during cognitive load, i.e., in conditions closer to real life situations. Following a lesion of the nervous system affecting the transmission or processing of sensory input and motor output, future studies should examine whether performance of the grip-lift task becomes even more dependent on the availability of cognitive resources. Most importantly, assessing motor performance under cognitive load might be a more sensitive mean to actually assess motor abilities and its impact in real life situations.

## Author Contributions

EG, AM, J-LT and VL: conceived and designed the experiments; analyzed the data; wrote the manuscript. EG: performed the experiments.

## Conflict of Interest Statement

The authors declare that the research was conducted in the absence of any commercial or financial relationships that could be construed as a potential conflict of interest.

## Footnotes

^1^http://www.vislab.ucl.ac.uk/cogent_2000
